# “Mitolocalization”: A Novel Marker of Mitochondrial Quality Control?

**DOI:** 10.1016/j.chstcc.2024.100073

**Published:** 2024-04-12

**Authors:** Bryan D. Kraft

**Affiliations:** Division of Pulmonary and Critical Care Medicine, Department of Medicine, Washington University School of Medicine.

Over the last 2 decades, it has been increasingly evident that metabolic impairment and mitochondrial injury are key drivers of sepsis-induced acute organ failure.^1,2^ Mitochondrial damage is evident as early as 12 h after sepsis onset, characterized histologically by mitochondrial swelling, fragmentation, and vacuolization,^3,4^ and molecularly by impaired oxidative phosphorylation.^3,5^ The host response to mitochondrial damage is activation of mitochondrial biogenesis, a highly coordinated program involving transcriptional activation of both nuclear and mitochondrial genes to regenerate new healthy mitochondria and restore mitochondrial volume density.^6^ Damaged or dysfunctional mitochondria that produce excessive reactive oxygen species are recycled via mitophagy, an autophagic lysosome-mediated selective degradation program.^7,8^ Both programs together constitute mitochondrial quality control (MQC).^2^ Early activation of mitochondrial biogenesis in patients with sepsis, as measured by higher messenger RNA levels of transcription factors and mitochondrial DNA copy numbers, has been previously associated with higher survival and ICU-free days,^1,3^ and mechanistically linked to activation of the antioxidant and counterinflammatory host responses.^9^ To date, the mitochondrial biogenesis program has been interrogated in human patients with sepsis in several tissues, including lung,^10^ skeletal muscle,^3^ and peripheral blood mononuclear cells (PBMCs).^1^ Peripheral blood is ideal because it is an easily retrievable tissue that can be collected serially; however, whether peripheral blood cells (and which cell types in particular) are representative of the other vital organs has been controversial.

In this issue of *CHEST Critical Care*, Thon et al^11^ interrogated the mitochondrial biogenesis program in 75 patients with sepsis by performing a proximity ligation assay to measure the interactions of two proteins, mitochondrial transcription factor A (TFAM) and mitochondrial transcription factor B2 (TF2BM), in situ in PBMCs. TFAM and TF2BM are both nuclear-encoded mitochondrial transcription factors that localize to the mitochondrial genome, bind to mitochondrial RNA polymerase to form the mitochondrial transcription initiation complex, and begin replication of mitochondrial DNA, an early step toward mitochondrial biogenesis. Building on prior work that found depleted intramitochondrial TFAM levels despite higher total TFAM expression^12^ (suggesting TFAM may be maldistributed within the cell), the authors used a proximal ligation assay that uses a fluorescent reporter to determine when both proteins are in close proximity to each other forming the transcription initiation complex. The TFAM-TF2BM interactions were counted in PBMCs collected on study days 1 and 4 and compared with interaction counts of 22 healthy control patients and 13 nonseptic critically ill patients. The counts were then correlated with survival and degree of organ failure as measured by the Sequential Organ Failure Assessment score. They also measured messenger RNA levels of several other key regulators of mitochondrial biogenesis (eg, nuclear respiratory factor 1) and mitochondrial DNA copy number. The authors found several interesting findings. First, they confirmed prior observations that transcriptional activation of mitochondrial biogenesis is evident as early as study day 1 in patients with sepsis and septic shock. Second, they showed that PBMC mitochondrial DNA copy number was lower in nonsurvivors than in survivors, supporting the overarching hypothesis that mitochondrial replication after mitochondrial loss is vital to recovery from and survival of septic illness. Third, the TFAM-TF2BM protein interactions in PBMCs correlated with Sequential Organ Failure Assessment score, suggesting this novel measure in peripheral blood may in fact be reflective of other vital organs and sepsis illness severity in general. Finally, after adjusting for baseline variables and SOFA score, higher TFAM-TF2BM counts on day 4 and a significant fold change from baseline were strongly associated with survival. Higher interaction counts were also significantly associated with sepsis recovery (ie, ICU freedom). Interestingly, and in contrast to other studies, this study did not find that messenger RNA levels of certain mitochondrial biogenesis transcription factors or coactivators were associated with survival or ICU freedom. The reason(s) for this discrepancy is not immediately clear; however, there were differences in definitions of study cohorts and time points used across the different studies which may make comparisons difficult.

Nevertheless, the authors’ use of a proximity ligation assay to measure TFAM-TFB2M protein interactions is a novel potential method to interrogate MQC in patients with sepsis, and their findings are supportive of their hypothesis that deranged TFAM localization to the mitochondria (despite higher protein expression) may be another factor that impairs activation of mitochondrial biogenesis.^12^ However, the study raises further questions about the biological implications of possible TFAM maldistribution that deserve further study. First, to what extent is the TFAM-TFB2M protein interaction confounded by sepsis-induced mitochondrial injury leading to a reduction in mitochondrial copy number? Although it is apparent that the two proteins are found in some but not all mitochondria at a given time, it seems plausible that the overall reduction in mitochondrial mass during sepsis due to mitochondrial damage will (at least somewhat) confound the measurement of the two proteins interacting. Second, it is not known whether the TFAM-TF2BM protein interactions are being measured in healthy mitochondria, damaged mitochondria, or both. This is important because future interventions would strive to maximize TFAM-TF2BM localization to functional mitochondria to start mitochondrial DNA replication, but not to dysfunctional or damaged ones that require selective degradation. Future studies should investigate whether there are qualitative differences in the mitochondrial function in those where the two proteins colocalize compared with those where they do not. Third, what are the factors at play that regulate localization (homing) of TFAM and TF2BM to mitochondria, and how and why does this process break down? Finally, are there other transcription factors or regulators that are also missing in action that could be equally or more important in this potential localization deficit?

Overall, the authors’ study raises important questions of whether transcriptional activation alone is enough to induce mitochondrial biogenesis, or whether defective localization of transcription factors (eg, TFAM, TF2BM) represents an additional lesion in MQC during sepsis. If the authors are correct, then impaired “mitolocalization” of TFAM and TF2BM may represent a novel component of MQC during sepsis that is worthy of further study.
